# The association between plasma metabolites and sleep quality in the Southall and Brent Revisited (SABRE) Study: A cross‐sectional analysis

**DOI:** 10.1111/jsr.13245

**Published:** 2020-12-06

**Authors:** Constantin‐Cristian Topriceanu, Therese Tillin, Nishi Chaturvedi, Roshni Joshi, Victoria Garfield

**Affiliations:** ^1^ University College London Medical School London UK; ^2^ Department of Population Science and Experimental Medicine Institute of Cardiovascular Science University College London London UK; ^3^ MRC Unit for Lifelong Health and Ageing University College London London UK

**Keywords:** amino acids, difficulty falling asleep, lipoproteins, metabolites, snoring

## Abstract

We examined the association between plasma metabolites and abnormal sleep patterns using data from the Southall and Brent REvisited (SABRE) cohort. Nuclear magnetic resonance spectroscopy provided 146 circulating plasma metabolites. Sleep questionnaires identified the presence or absence of: difficulty falling asleep, early morning waking, waking up tired, and snoring. Metabolites were compared between the sleep quality categories using the *t* test, and then filtered using a false discovery rate of 0.05. Generalised linear models with logit‐link assessed the associations between filtered metabolites and sleep phenotypes. Adjustment was made for important demographic and health‐related covariates. In all, 2,718 participants were included in the analysis. After correcting for multiple testing, three metabolites remained for difficulty falling asleep, 59 for snoring, and none for early morning waking and waking up tired. After adjusting for sex, age, ethnicity and years of education, 1 standard deviation increase in serum histidine and valine associated with lower odds of difficulty falling asleep by 0.89–0.90 (95% confidence intervals [CIs] 0.80–0.99). Branched‐chain and aromatic amino acids (odds ratios [ORs] 1.19–1.25, 95% CIs 1.09–1.36) were positively associated with snoring. Total cholesterol in low‐density lipoprotein (OR 0.90, 95% CI 0.83–0.97) and high‐density lipoprotein (OR 0.88, 95% CI 0.81–0.95) associated with lower odds of snoring. In the fully adjusted model, most associations persisted. To conclude, histidine and valine associated with lower odds of difficulty falling asleep, while docosahexaenoic acid and cholesterol in low‐density lipoprotein and high‐density lipoprotein subfractions associated with lower odds of snoring. Identified metabolites could provide guidance on the metabolic pathways associated with adverse sleep quality.

## 1 INTRODUCTION

Sleep is a vital component of the human circadian rhythm. Abnormal sleep patterns are increasingly common. Two of the most prevalent sleep disorders are insomnia and snoring, with recent estimates suggesting insomnia is reported in >30% (LeBlanc et al., 2007) and snoring in >20% of the global population (Enright et al., 1996).

Sleep is of primordial importance in physiological homeostasis. While adverse sleep phenotypes are associated with negative health consequences, including cardiovascular disease and cancer (Medic et al., 2017), the triggers and pathways that may contribute to abnormal sleep phenotypes and related detrimental health outcomes are still “hot” research topics.

Metabolic dysfunction has been previously associated with sleep phenotypes (Depner et al., 2014). Insomnia and short sleep duration have been associated with increased odds of developing type 2 diabetes mellitus (T2DM) (Vgontzas et al., 2009), which could be mediated by branched‐chain amino acids (BCAAs). Snoring has been associated with disordered metabolic processes including insulin resistance, hyperglycaemia and dyslipidemia (high triglycerides, high low‐density lipoprotein [LDL] cholesterol, and low high‐density lipoprotein [HDL] cholesterol) (Alexopoulos et al., 2011; Sharma & Kavuru, 2010). However, the directionality of the association between metabolites and sleep phenotypes is still unclear. Although epidemiological studies postulate that disordered sleep may be both a cause and a consequence of abnormal metabolism, any potential causal effects relating to these claims are still to be elucidated.

Thus, using a metabolomics approach (Fabian et al., 2013) could lead to a better understanding of the mechanisms underlying abnormal sleep phenotypes, as well as provide a direction for future novel guided therapies. Similar approaches have been successfully employed, e.g. in characterising novel predictors for heart failure (Delles et al., 2018).

In the present cross‐sectional study, nuclear magnetic resonance (NMR) spectroscopy was used to identify metabolites associated with adverse sleep phenotypes (difficulty falling asleep, early morning waking, waking up tired, and snoring) reported in participants from the Southall and Brent REvisited (SABRE) cohort.

## 2 METHODS

### 2.1 Participants and study design

The SABRE study is a tri‐ethnic cohort of European, South Asian and African Caribbean participants living in West and North London (Southall and Brent districts). Between 1988 and 1991, participants aged 40–69 years were randomly selected from 5‐year age and sex stratified primary care lists (*n* = 4,063) and workplaces (*n* = 795). The full cohort details have been published elsewhere (Tillin et al., 2012). Ethnicity was self‐assigned and agreed with the interviewer. South Asians and African Caribbeans were all first‐generation migrants.

At baseline, participants were invited to a clinic appointment, which involved completing a health and lifestyle questionnaire that included questions on sleep patterns. Fasting bloods were collected, and anthropometrics and blood pressure were measured. Diabetes was identified from self‐report of physician diagnosis or receipt of anti‐diabetes medications. In addition, oral glucose tolerance testing was performed. Undiagnosed diabetes was identified retrospectively using World Health Organization (WHO) 1999 criteria (Alberti & Zimmet, 1998).

The SABRE baseline study was granted ethics approval from Ealing, Hounslow and Spelthorne, Parkside, and University College London Research Ethics Committees.

### 2.2 Exposures

The exposures were 146 NMR measured circulating plasma metabolites including amino acids, small molecules (e.g. glycerol, pyruvate) and a detailed lipid profile consisting of 16 lipoprotein subclasses together with their lipid component concentrations (total cholesterol, cholesterol‐esters, free cholesterol, total lipids, phospholipids, and triglycerides) and particle dimensions (i.e. diameter).

Serum fasting samples were obtained from 3,700 participants (from the Southall Centre only) at baseline and were stored at −80°C. In 2012, a proton NMR spectrum was employed to detect circulating plasma metabolite levels following the signal suppression of other molecules according to methodologies previously described (Soininen et al., 2009; Würtz et al., 2017). Ratios of metabolites (e.g. triglycerides to phosphoglycerides) were excluded as they were beyond the scope of our study.

None of the study participants was receiving lipid‐lowering medication at the time of the baseline studies.

### 2.3 Outcomes

The outcomes of interest were four sleep quality phenotypes: difficulty falling asleep, early morning waking, waking up tired, and snoring.

Participants were asked to rate their sleep quality in the past 30 days at visit 1 (1988–90) using four questions adapted from the validated Jenkins Sleep Questionnaire (JSEQ) (Jenkins et al., 1988). They were asked whether they felt they had difficulties falling asleep at night, had been waking up too early in the morning, whether they woke up feeling tired or had problems with snoring during the night. All sleep phenotypes were rated binary (i.e. “No”/0 or “Yes”/1).

### 2.4 Covariates

Covariates were selected a priori based on prior associations with sleep quality and metabolism. Covariates were recorded at the time of the baseline studies (when serum samples were collected for storage) and included: age, sex, ethnicity, years of education, waist–hip ratio (WHR), body mass index (BMI), cardiovascular disease (CVD; coronary artery disease and stroke), T2DM, hypertension medication, total number of alcohol units per week, and smoking status (never smoked, ex‐smoker, current smoker). CVD, T2DM and hypertension medication were recorded as binary (i.e. “No”/0 or “Yes”/1).

### 2.5 Statistical methods

Data distribution was assessed graphically using histograms and statistically using the Shapiro–Wilk test. Continuous variables were expressed as medians (interquartile ranges), while categorical variables were expressed as counts (percentage).

Metabolite concentrations were log‐transformed, mean centred and scaled to a standard deviation (*SD*) of 1 before further analysis.

A *t* test analysis was used as a screening tool to identify metabolites linked with sleep phenotypes, correcting for multiple testing at a false discovery rate (FDR) of 0.05 (Benjamini & Hochberg, 1995). Metabolites that passed this threshold were further referred to as “candidate metabolites”. Candidate metabolite associations with sleep phenotypes were evaluated using multivariable generalised linear models (GLMs) accounting for age, sex, ethnicity, and years of education (Model 1). Further adjustments were Model 1 plus WHR, CVD, T2DM, anti‐hypertensive medication, alcohol units, and smoking status (Model 2). As BMI captures a somewhat different profile of excess weight as compared to the WHR, an additional Model 2 where BMI was used instead of WHR was fitted. Where multiple metabolites were associated with a sleep quality phenotype, a Manhattan plot was used for visual representation. In the Manhattan plot, –log10(*p* values) were plotted on the *y*‐axis, while the *x*‐axis consisted of metabolites grouped into relevant categories.

About a fifth of all participants reported zero weekly alcohol intakes. To account for this high degree of zero‐inflation (He et al., 2014), a modified version of GLM (using template model builder [glmmTMB]) was chosen (Appendix S1).Thus, glmmTMB with binomial distribution and logit link (equivalent to logistic regression) using metabolites as exposures was employed to predict the binary sleep phenotypes as outcomes. As snoring was associated with HDL and LDL in the same direction, a Pearson’s correlation analysis was performed between HDL and LDL subfractions.

The *t* test is not able to discriminate between groups where minor differences exist. This may not identify biologically relevant metabolites (Fabian et al., 2013). Thus, we ran the regression models for all the available metabolites as a sensitivity analysis.

As sleep health is multi‐dimensional (Buysse, 2014), we created a composite sleep score. First, we performed a principal component analysis (PCA) of the included sleep phenotypes. For each principal component, the weights were normalised so that they added up to 1. As the first principal component accounts for most variability in the indicators (Hosseini et al., 2019), we constructed a PCA‐weighted sleep composite score (wSleep) as the weighted average of the sleep phenotypes using the first principal weights. As the sleep composite score was discrete rather than continuous, GLMs with Poisson distribution and log link assessed the associations between metabolites and wSleep (Gardner et al., 1995). Regression results were then filtered at a FDR of 0.05.

Statistical analysis was performed in R, version‐3.6.0 (R Foundation for Statistical Computing, Vienna, Austria), with *p* < .05 considered statistically significant.

## 3 RESULTS

### 3.1 Participant characteristics

Of the 4,858 SABRE participants, 3,700 (from the Southall Centre only) had stored blood samples with 3,255 being viable for NMR analysis. Furthermore, 2,718 had all sleep phenotypes, covariates, and metabolites. Of the 2,718 included participants, 2,379 (87.53%) were male and 664 (24.43%) were current smokers. The mean (*SD*) age of the cohort was 52.10 (7.18) years and the mean (*SD*) WHR was 0.95 (0.08). Difficulty falling asleep was reported in 453 (16.67%) participants, early morning waking in 1,108 (40.77%), waking up tired in 927 (34.11%), and snoring in 1,051 (38.67%). Participant characteristics are summarised in Table 1.

**Table 1:** Baseline demographic characteristics of Southall And Brent Revisited (SABRE) cohort participants stratified by sleep phenotype status

Sleep phenotype	Difficulty falling asleep			Early morning waking			Waking up tired			Snoring			Overall
	Yes 453 (16.67%)	No 2,265 (83.33%)	*p*	Yes 1,108 (40.77%)	No 1,610 (59.23%)	*p*	Yes 927 (34.11%)	No 1,791 (65.89%)	*p*	Yes 1,051 (38.67%)	No 1,667 (61.33%)	*p*	*n* = 2,718
Age, years, mean (*SD*)	52.08 (7.27)	52.10 (7.16)	.966	52.15 (7.11)	52.06 (7.23)	.709	51.25 (7.05)	52.53 (7.20)	**<.0001**	52.66 (7.01)	51.74 (7.25)	**.001**	52.10 (7.18)
Male sex, *n* (%)	361 (79.69)	2018 (89.09)	**<.0001**	961 (86.73)	1,418 (88.07)	.327	787 (84.89)	1592 (89.06)	**.004**	982 (93.43)	1,397 (83.80)	**<.0001**	2,379 (87.53)
Ethnicity, *n* (%)													
European	193 (42.60)	1,159 (51.17)	**.002**	468 (42.24)	884 (54.91)	**<.0001**	456 (49.19)	896 (50.03)	.528	512 (49.57)	840 (50.39)	.681	1,352 (49.74)
South Asian	238 (52.5)	984 (43.44)		591 (53.34)	631 (39.19)		427 (46.06)	795 (44.39)		481 (45.77)	741 (44.45)		1,222 (44.96)
African Caribbean	22 (0.05)	122 (5.39)		49 (4.42	95 (5.9)		44 (4.75)	100 (5.58)		58 (4.66)	86 (5.16)		144 (5.30)
Years of education, mean (*SD*)	10.97 (3.25)	11.25 (3.11)	.083	11.11 (3.18)	11.27 (3.11)	.197	11.14 (3.27)	11.24 (3.07)	.461	11.07 (3.19)	11.29 (3.11)	.062	11.21 (3.14)
Waist–hip ratio, mean (*SD*)	0.94 (0.09)	0.94 (0.08)	.864	0.95 (0.08)	0.94 (0.08)	**.0004**	0.94 (0.09)	0.95 (0.08)	.546	0.96 (0.08)	0.93 (0.09)	**<.0001**	0.95 (0.08)
Cardiovascular disease, *n* (%)	59 (13.02)	184 (8.12)	**.001**	119 (10.74)	124 (7.70)	**.008**	112 (12.08)	131 (7.31)	**<.0001**	114 (10.85)	129 (7.74)	**.007**	243 (8.94)
Diabetes, *n* (%)	72 (15.89)	276 (12.19)	**.038**	151 (13.63)	187 (11.62)	.313	120 (12.95)	228 (12.73)	.922	152 (14.46)	196 (11.76)	**.046**	348 (12.80)
Anti‐hypertensives, *n* (%)	80 (17.66)	279 (12.32)	**.003**	171 (15.43)	188 (11.68)	**.005**	149 (16.07)	210 (11.72)	**.002**	165 (15.70)	194 (11.63)	**.003**	359 (13.21)
Alcohol consumption, units, mean (*SD*)	12.14 (17.63)	11.67 (15.24)	.601	11.94 (15.56)	11.62 (15.01)	.610	12.15 (16.58)	11.54 (15.16)	.351	12.71 (16.78)	11.14 (14.88)	**.013**	11.75 (15.66)
Smoking status, *n* (%)													
Never	231 (50.99)	1,211 (53.47)	**.008**	622 (56.14)	820 (50.93)	**.027**	482 (52.00)	960 (48.71)	.066	518 (49.29)	924 (55.43)	**.006**	1,442 (53.05)
Ex‐smoker	96 (21.19)	568 (25.08)		231 (20.85)	381 (23.66)		232 (25.00)	380 (21.22)		250 (23.79)	362 (21.72)		612 (22.52)
Current smoker	126 (27.82)	486 (21.45)		255 (23.01)	409 (25.41)		213 (23.00)	451 (25.18)		283 (26.92)	381 (22.85)		664 (24.43)

Characteristics of participants stratified by sleep phenotypes. Values are presented as mean (*SD*) for continuous variables or *n* (%) for categorical variables. The *p* values were calculated using *t* test for continuous variables or chi‐squared test for categorical variables. Significant *p* values are highlighted in **bold**.

### 3.2 Screening for candidate metabolites

Of the 146 available metabolites, 12 metabolites were identified for difficulty falling asleep, four for early morning waking, two for waking up tired, and 73 for snoring using the *t* test (Supplementary Table S1). After correcting for multiple testing at a FDR of 0.05, histidine, leucine and valine remained as candidate metabolites for difficulty falling asleep. In addition, 59 metabolites were further considered as candidates in association with snoring. Lastly, none remained for early morning waking and waking up tired (Supplementary Table S1).

### 3.3 Difficulty falling asleep

After adjusting for age, sex, ethnicity and years of education (Model 1), serum histidine and valine were inversely associated with difficulty falling asleep as follows: 1 *SD* increases in serum histidine and valine were associated with lower odds of difficulty falling asleep with an odds ratio (OR) of 0.89 (95% confidence interval [CI] 0.80–0.99) and 0.90 (95% CI 0.81–0.99), respectively. Associations persisted after further adjustment for covariates in Model 1 plus WHR, CVD, T2DM, anti‐hypertensive medication, alcohol units, and smoking status (Model 2) (Supplementary Table S2). When we used BMI instead of WHR, the results were similar (Supplementary Table S3). Individuals experiencing difficulty falling asleep had lower plasma levels of histidine and valine (Supplementary Table S4).

### 3.4 Snoring

**Figure 1:**
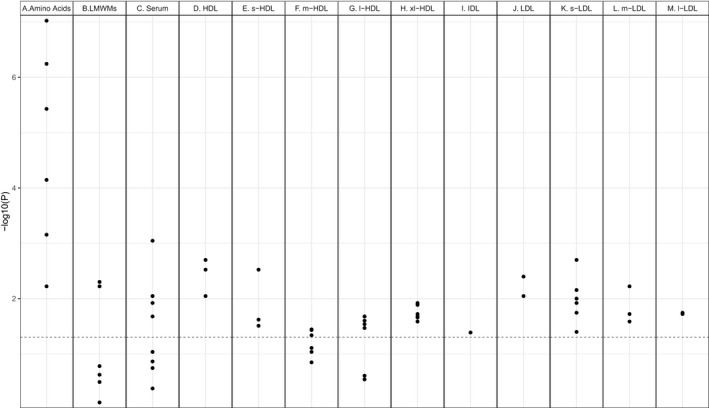
Manhattan plot of the associations between plasma metabolites and snoring. Metabolites with a significant association with snoring in Model 1 are annotated. The red line corresponds to the significance level of .05. HDL, high‐density lipoprotein; s‐HDL, small‐HDL; m‐HDL, medium‐HDL; l‐HDL, large‐HDL, xl‐HDL, extra‐large HDL; IDL, intermediate‐density lipoprotein; LDL, low‐density lipoprotein; s‐LDL, small‐LDL; m‐LDL, medium‐LDL; l‐LDL, large‐LDL; CE, cholesterol‐esters; FC, free cholesterol

**Figure 2:**
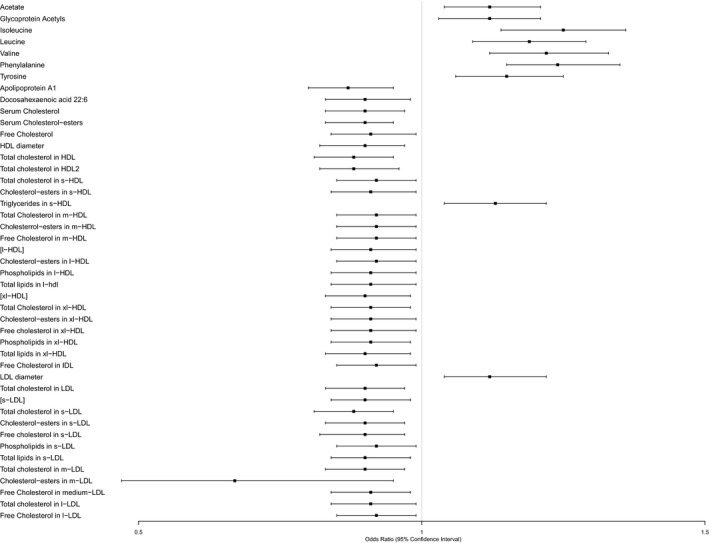
Forest plot of the ORs and 95% CIs for the association between plasma metabolites and snoring which were significant in Model 1 (adjusted for age, sex, ethnicity and years of education). OR, odds ratio; CI, confidence interval; HDL, high‐density lipoprotein; s‐HDL, small‐HDL; m‐HDL, medium‐HDL; l‐HDL, large‐HDL, xl‐HDL, extra‐large HDL; IDL, intermediate‐density lipoprotein; LDL, low‐density lipoprotein; s‐LDL, small‐LDL; m‐LDL, medium‐LDL; l‐LDL, large‐LDL; CE, cholesterol‐esters; FC, free cholesterol

Of the 59 candidate metabolites, 45 remained associated with snoring in Model 1 (Figure 1). The ORs associated with 1 *SD* increments in each of the 45 metabolites in Model 1 are presented in Table 2 and visually illustrated in Figure 2. Model 2 results are presented in Supplementary Table S2.

**Table 2:** Associations between the metabolites who have passed the screening stage and the sleep phenotypes

Sleep phenotype	Metabolite	Multivariable adjusted OR (95% CI)	*p*
Difficulty falling asleep	Histidine	0.89 (0.80–0.99)	**.031**
	Leucine	0.92 (0.82–1.03)	.148
	Valine	0.90 (0.81–0.99)	**.045**
Snoring	Acetate	1.12 (1.04–1.21)	**.005**
	Creatinine	1.01 (0.93–1.11)	.754
	Glucose	1.04 (0.96–1.13)	.322
	Glutamine	0.94 (0.87–1.01)	.106
	Glycoprotein acetyls	1.12 (1.03–1.21)	**.006**
	Isoleucine	1.25 (1.14–1.36)	**<.0001**
	Lactate	1.05 (0.97–1.14)	.238
	Leucine	1.19 (1.09–1.29)	**<.0001**
	Phosphatidylcholine	0.95 (0.88–1.03)	.180
	Phenylalanine	1.24 (1.15–1.35)	**<.0001**
	Tyrosine	1.15 (1.06–1.25)	**.0007**
	Valine	1.22 (1.12–1.33)	**<.0001**
	Total cholines	0.95 (0.87–1.02)	.166
	Apolipoprotein A‐I	0.87 (0.80–0.95)	**.0009**
	Docosahexaenoic acid 22:6	0.90 (0.83–0.98)	**.012**
	Linoleic acid 18:2	0.94 (0.87–1.02)	.137
	Polyunsaturated fatty acids	0.93 (0.86–1.01)	.092
	Serum cholesterol	0.90 (0.83–0.97)	**.009**
	Serum cholesterol‐esters	0.90 (0.83–0.95)	**.009**
	Free cholesterol	0.91 (0.84–0.99)	**.021**
	Serum triglycerides	1.03 (0.95–1.12)	.421
	Total cholesterol in HDL	0.88 (0.81–0.95)	**.002**
	Total cholesterol in HDL2	0.88 (0.82–0.96)	**.003**
	HDL diameter	0.90 (0.82–0.97)	**.009**
	Total cholesterol in small HDL	0.92 (0.85–0.99)	**.031**
	Cholesterol‐esters in small HDL	0.91 (0.84–0.99)	**.024**
	Triglycerides in small HDL	1.13 (1.04–1.22)	**.003**
	Total cholesterol in medium HDL	0.92 (0.85–0.99)	**.036**
	Cholesterol‐esters in medium HDL	0.92 (0.85–0.99)	**.037**
	Free cholesterol in medium HDL	0.92 (0.85–0.99)	**.046**
	Total lipids in medium HDL	0.93 (0.86–1.01)	.078
	Concentration of medium HDL particle	0.93 (0.86–1.01)	.092
	Phospholipids in medium HDL	0.94 (0.87–1.02)	.142
	Total cholesterol in large HDL	0.95 (0.85–1.05)	.289
	Cholesterol‐esters in large HDL	0.92 (0.85–0.99)	**.034**
	Free cholesterol in large HDL	0.95 (0.87–1.04)	.248
	Total lipids in large HDL	0.91 (0.84–0.99)	**.025**
	Concentration of large HDL particle	0.91 (0.84–0.99)	**.029**
	Phospholipids in large HDL	0.91 (0.84–0.99)	**.021**
	Total cholesterol in very large HDL	0.91 (0.84–0.98)	**.019**
	Cholesterol‐esters in very large HDL	0.91 (0.84–0.99)	**.022**
	Free cholesterol in very large HDL	0.91 (0.84–0.99)	**.026**
	Total lipids in very large HDL	0.90 (0.83–0.98)	**.012**
	Concentration of very large HDL particle	0.90 (0.83–0.98)	**.013**
	Phospholipids in very large HDL	0.91 (0.84–0.98)	**.021**
	Free cholesterol in IDL	0.92 (0.85–0.99)	**.041**
	Total cholesterol in LDL	0.90 (0.83–0.97)	**.009**
	LDL diameter	1.12 (1.04–1.22)	**.004**
	Total cholesterol in small LDL	0.88 (0.81–0.95)	**.002**
	Cholesterol‐esters in small LDL	0.90 (0.83–0.97)	**.007**
	Free cholesterol in small LDL	0.90 (0.82–0.97)	**0.010**
	Total lipids in small LDL	0.90 (0.84–0.98)	**.012**
	Concentration of small LDL particle	0.91 (0.84–0.98)	**.018**
	Phospholipids in small LDL	0.92 (0.85–0.99)	**.040**
	Total cholesterol in medium LDL	0.90 (0.83–0.97)	**.006**
	Cholesterol‐esters in medium LDL	0.67 (0.47–0.95)	**.026**
	Free cholesterol in medium LDL	0.91 (0.84–0.98)	**0.019**
	Total cholesterol in large LDL	0.91 (0.84–0.99)	**.018**
	Free cholesterol in large LDL	0.92 (0.85–0.99)	**.038**

All analyses reported here consisted of generalised linear mixed models with binomial distribution and logit link (i.e. logistic regression). Model 1 was adjusted for age, sex, ethnicity and years of education. Model 2 results are presented in Supplementary Table S2. Significant *p* values are highlighted in **bold**.

OR, odds ratio; CI, confidence interval; HDL, high‐density lipoprotein; IDL, intermediate‐density lipoproteins; LDL, low‐density lipoprotein.

The BCAAs (isoleucine, leucine and valine) and aromatic amino acids (phenylalanine and tyrosine) were associated with greater odds of snoring (ORs in the range of 1.19 to 1.25). Similarly, acetate and glycoprotein acetyls associated with 1.12 (95% CI 1.03–1.21) higher odds of snoring. In Model 2, associations persisted for all except glycoprotein acetyls and tyrosine (Supplementary Table S2).

After adjusting for Model 1, serum apolipoprotein A‐I (OR 0.87, 95% CI 0.80–0.95) and docosahexaenoic acid (DHA; OR 0.90, 95% CI 0.83–0.98) were inversely associated with snoring. Total cholesterol in HDL (OR 0.88, 95% CI 0.81–0.95) and in LDL (OR 0.90, 95% CI 0.83–0.97) appeared to be beneficial. Regarding the subfraction breakdown, total cholesterol in small, medium and very‐large HDL (ORs 0.91–0.92, 95% CI 0.84–0.99) and in small, medium and large LDL (ORs 0.88–0.91 95% CI 0.81–0.99) were associated with lower odds of snoring. In addition, cholesterol‐esters, free cholesterol, phospholipids and total lipids from certain LDL and HDL subfractions were also inversely associated with snoring (Figure 2), with the lowest OR for lower odds of snoring being observed for cholesterol‐esters in medium‐LDL (OR 0.67, 95% CI 0.47–0.95). The HDL and LDL subfractions were mostly positively correlated (Supplementary Figure S1). In Model 2, significant associations mostly persisted except for the very‐large HDL subfractions (Supplementary Table S2). When we used BMI instead of WHR, most associations persisted except for five metabolites (Supplementary Table S3). Individuals reporting snoring had higher plasma levels of BCAAs and aromatic amino acids. In addition, they had lower cholesterol in HDL and LDL (Supplementary Table S4).

### 3.5 Sensitivity analysis

Additional metabolites that were associated with the sleep phenotypes in the fully adjusted models (i.e. Model 2), but which did not pass the *t* test screening stage were identified (Supplementary Table S5). A 1 *SD* increase in glycine, free cholesterol and sphingomyelins was related to greater odds (ORs ≅ 1.15) of having difficulty falling asleep. Creatinine and valine were associated with lower odds (ORs ≅ 0.90) of early morning waking, while albumin and lactic acid associated with lower odds (ORS ≅ 0.87) of waking up tired. Free cholesterol, phospholipids and total lipids in medium and large LDL, small very LDL (VLDL) and very small VLDL; and apolipoprotein B and omega‐3 fatty acids were associated with lower odds of snoring (ORs ≅ 0.90).

### 3.6 Composite sleep score (wSleep)

The first principal weights corresponding to the sleep phenotypes were: 0.33 for difficulty falling asleep, 0.30 for early morning waking, 0.31 for waking up tired, and 0.06 for snoring. The regression results for wSleep (calculated as a weighted sum of the first principal weights) are presented in Supplementary Table S6. A unit increase in glycoprotein acetyls, sphingomyelins; and triglycerides in serum, small and medium LDL, and small, medium, large and very large VLDL associated with 3%–4% (95% CIs 0%–8%) higher wSleep scores. None remained after filtering at a FDR of 0.05. The second principal weights corresponding to the sleep phenotypes were: 0.17 for difficulty falling asleep, 0.02 for early morning waking, 0.06 for waking up tired, and 0.75 for snoring.

## 4 DISCUSSION

Using data from the SABRE cohort, we show in this cross‐sectional analysis that circulating plasma metabolites are associated with distinct sleep quality phenotypes. In particular, metabolites that were associated both with higher as well as lower odds of difficulty falling asleep and snoring were identified. In addition, increased levels of some of the metabolites were associated with lower odds of early morning waking and waking up tired.

Difficulty falling asleep is one of the most frequent symptoms reported by patients with insomnia (Lombardero et al., 2019). Our present results show that increased levels of histidine, isoleucine and valine were associated with lower odds of difficulty falling asleep, even after adjusting for the relevant covariates. Interestingly, all three are essential amino acids, which means that they cannot be synthesised de novo, and therefore, they may be linked to a deficient diet. Histidine is a precursor of histamine, which has been proposed as a regulator of wakefulness (Thakkar, 2011). Isoleucine and valine are precursors of both glutamate and gamma‐aminobutyric acid (GABA) (Sweatt et al., 2004), the main excitatory and inhibitory neurotransmitters. By restoring the inhibition:excitatory ratios, their supplementation could restore normal sleep patterns. In the present study, we report that BCAAs are associated with lower odds (OR ≅ 0.90) of difficulty falling asleep. To date, BCAAs such as leucine and valine have been successfully trialed to improve sleep quality in certain population groups, such as patients with cirrhosis (Ichikawa et al., 2010).

We also observed that in SABRE snoring was associated with 31 metabolites, suggesting a potential complex metabolic disturbance. In addition, the PCA revealed that snoring could potentially be a sleep dimension in itself. Most of these metabolites are from the lipid profile and include serum lipid extracts (such as DHA, polyunsaturated fatty acids) and cholesterol, cholesterol‐esters and phospholipids in small/medium/large LDL and HDL fractions. Although the snoring–insulin resistance–dyslipidaemia connection has previously been reported (Alexopoulos et al., 2011), the novelty of the present study comes from the detailed lipoprotein analysis that identified specific lipoprotein components. Our present data show that both higher LDL and HDL associate with lower odds of snoring. The effect of HDL is consistent with the existing literature (Alexopoulos et al., 2011), but the effect of LDL is not. Although an inverse correlation between HDL and LDL is to be expected (Supplementary Figure S1), it should be noted that lipoproteins in the metabolism are in a constant state of flux with complex interactions, rather than discrete measures. In addition, although LDL‐cholesterol is considered to be the most atherogenic, there is no robust evidence to suggest it has a negative impact on sleep quality.

**Figure 3:**
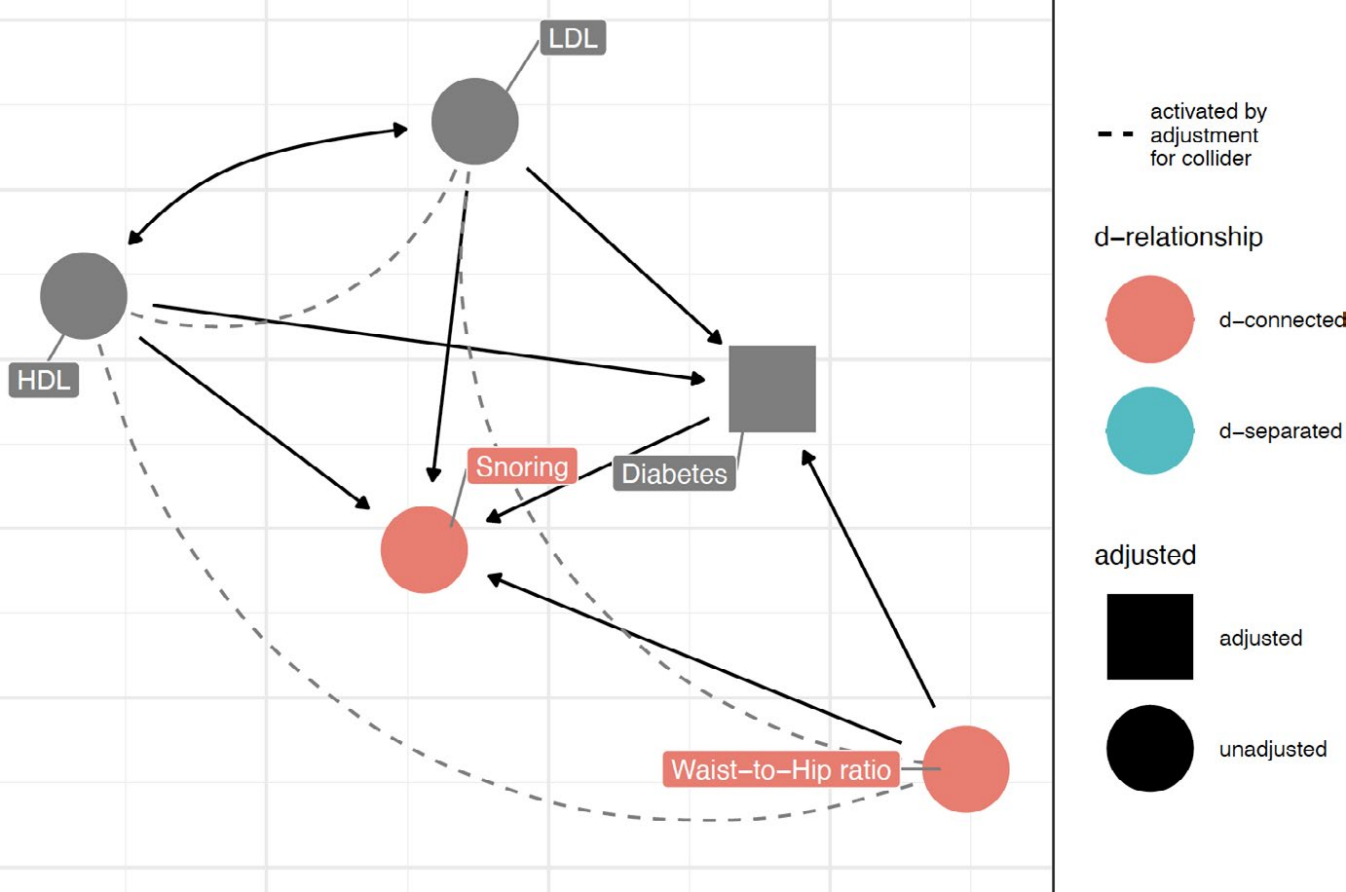
Directed acyclic graph for snoring. Low HDL, high LDL, high waist–hip ratio (WHR) and type 2 diabetes mellitus (T2DM) have been shown to be associated with snoring. However, a high WHR is associated with T2DM and both variables are associated with dyslipidaemia. In addition, low HDL and high LDL are also common in T2DM. This directed acyclic graph highlights the complex interactions which exist within this system. HDL, high‐density lipoprotein; s‐HDL, small‐HDL; m‐HDL, medium‐HDL; l‐HDL, large‐HDL, xl‐HDL, extra‐large HDL; IDL, intermediate‐density lipoprotein; LDL, low‐density lipoprotein; s‐LDL, small‐LDL; m‐LDL, medium‐LDL; l‐LDL, large‐LDL; CE, cholesterol‐esters; FC, free cholesterol

The directionality of the associations between snoring, T2DM, WHR and metabolites is still a matter of debate as complex metabolic interactions exist (Figure 3). Lastly, the inverse association between DHA and snoring severity has previously been reported (Ladesich et al., 2011). Beneficial effects of ω‐3 fatty acids in terms of reduced daytime sleepiness have been observed after supplementation in deployed soldiers (Dretsch et al., 2014). DHA is an important component of neural membranes, which has been postulated to be both a synaptic and a neuromodulator altering the levels of glutamate, monoamines, acetylcholine, and endocannabinoids in the brain (Tanaka et al., 2012).

All BCAAs and phenylalanine were found to be associated with snoring. Phenylalanine is a precursor to dopamine and noradrenaline, which have been shown to be downregulated in sleep deprivation (Volkow et al., 2012). Interestingly, higher valine has been associated with snoring (higher odds), early morning waking (lower odds), and difficulty falling asleep (lower odds) making it more likely to be a key biological player in regulating sleep. Given the wide‐ranging effects, it could be that valine operates within a narrow homeostatic window with higher levels promoting sleep (lower odds of difficulty falling asleep and early waking), but if they are too high snoring could arise. This theory is supported by its involvement in multiple important processes, such as protein synthesis, energy production, glutamate compartmentalisation, and indirect control of serotonin, dopamine, and noradrenaline neurotransmitter synthesis (Fernstrom, 1539S). Lastly, as acetate generates acetyl coenzyme A, it has been proposed as an epigenetic metabolite to regulate lipid synthesis under hypoxia (Xue et al., 2016), which can occur during snoring.

Recently, metabolomics‐based risk scores have been developed to predict obstructive sleep apnea (OSA) (Ferrarini et al., 2013; Lebkuchen et al., 2018; Xu et al., 2016). Similarly, we also identified: isoleucine and valine, but in our present study, lactate, sphingomyelins and phosphatidyl cholines were not associated with snoring. The remainder of the metabolites were not available in our present data.

Our sensitivity analysis revealed that higher creatinine and valine were associated with lower odds of early morning waking. A positive relationship between creatinine and long sleep duration has been previously reported (Choi et al., 2017). Creatinine is a breakdown product of creatine phosphate. The latter is of neurophysiological importance acting as an antioxidant, a neuromodulator [of GABA A (GABA_A_) and of N‐methyl‐*D*‐aspartate (NMDA) receptors] and a regulator of neuronal energy metabolism. It has been postulated to neutralise the negative effects of reactive oxygen species that occur on the background of chronic psychological stress (Allen, 2012). To date, it has been successfully trialed to improve mood and performance after sleep deprivation (McMorris et al., 2006). Its potential for the prevention of early morning waking has not been yet explored in clinical studies. However, as our present cohort consisted mostly of middle‐aged individuals, poor kidney function may introduce confounding bias for the observed differences in creatinine levels between those who reported snoring and those who did not.

Waking up tired has been associated with chronic fatigue syndrome. Our sensitivity analysis identified that higher albumin and lactic acid are related to lower odds of waking up tired. Lower albumin correlates with fatigue in patients with chronic kidney disease (Jhamb et al., 2014). We are the first to generalise this association in a large‐scale, predominantly healthy cohort. Although we did not specifically measure tryptophan, the observed effect might be mediated by this albumin‐bound amino acid, as it has been linked to a higher serotonin:dopamine ratio leading to central, as opposed to peripheral fatigue (Meeusen, 2009). Serum lactate has previously been reported as a possible sleep/wake biomarker with higher levels during wakefulness; and persistent and sustained decline during non‐rapid eye movement sleep (Naylor et al., 2012). Serum lactate could potentially be extended as a biomarker for the early morning waking phenotype. It could also be that central fatigue may stem from a lower neuronal glucose consumption translating into lower plasma lactate levels.

There are a limited number of studies investigating the relationship between metabolites and sleep phenotypes. One such study in post‐menopausal women concluded that higher triglycerides are associated with poor sleep quality (Huang et al., 2019). Similarly, our present data show that higher triglycerides in serum or certain subfractions of LDL and VLDL were associated with higher wSleep scores.

The NMR quantification of serum metabolites offers a novel approach for the granular investigation of the molecular associations between a range of biomarkers and sleep phenotypes. However, our NMR database itself does not capture the whole metabolome, as it is mostly limited to amino acids, lipids, and small molecules. In addition, the present study also did not take advantage of the quantitative data (i.e. levels of metabolites) as they were log‐transformed, mean centred and scaled to a *SD* of 1 before further analysis. Lastly, metabolites are prone to biological variation and measurement error, which we were not able to assess. Such errors could bias the estimates presented and should be a consideration when evaluating the relationship between plasma metabolites and sleep phenotypes. We present associations in a tri‐ethnic UK cohort, but further analysis in similar cohorts would be required before reliably extending findings to wider, non‐UK populations. Another limitation was the long‐term storage of samples (>20 years) before NMR analysis.

The snoring phenotype is representative of a middle‐age cohort in terms of prevalence, which is highlighted by the relatively high number of associated metabolites passing a robust statistical analysis, most of which have been previously reported (Xu et al., 2016; Zhang et al., 2017). Although there are more objective tests to assess for adverse sleep phenotypes, especially for snoring (Arnardottir et al., 2016), our present study used self‐reported measures. Although females under‐report the prevalence of snoring, that is not the case for males (Westreich et al., 2019). However, difficulty falling asleep, early morning waking and waking up tired are more subjective and could be both under‐ and over‐reported (Bianchi et al., 2013; Landry et al., 2015; L. Zhang & Zhao, 2008). A limitation of wSleep is masking individual sleep quality phenotypes, each of which is capturing a different aspect of sleep. Moreover, it does not form a validated scale, as it contains only four questions from the JSEQ scale (Jenkins et al., 1988). In addition, there were only a few women included due to the study design (Tillin et al., 2012). As such, it is not surprising that difficulty falling asleep was under‐represented (Tang et al., 2017), occurring only in 17% of individuals.

Poor self‐reported sleep quality has also been suggested as an epiphenomenon for underlying mental health problems, such as depression and anxiety (Bower et al., 2010). Many of the metabolites we identified to be associated with our sleep phenotypes have previously been associated with depression (Bot et al., 2020; Huang et al., 2020). Examples of these include amino acids (e.g. valine, tyrosine etc.), small molecules (e.g. acetate, glycoprotein acetyls etc.) and lipids (e.g. cholesterol, triglycerides etc.). As mood‐related factors were not explored in the present study, our present results may reflect overlapping metabolic signatures between self‐reported sleep quality phenotypes and depression or anxiety.

Limitations to the present study include its inherent cross‐sectional nature and the failure to capture longitudinal effects or to support causality. The directionality of the associations between metabolites and sleep phenotypes is still a matter of debate and whether metabolites cause or are a consequence of abnormal sleep is yet to be elucidated.

## 5 CONCLUSION

Histidine and valine associated with lower odds of difficulty falling asleep, while BCAAs were positively associated with snoring. Total cholesterol in certain HDL and LDL subfractions appeared beneficial in terms of snoring. Although the present evidence is unable to support causality, the identified metabolites could provide a direction for future studies to further understand abnormal sleep patterns.

## CONFLICT OF INTEREST

The views expressed in this article are those of the authors who declare that they have no conflict of interest.

## AUTHOR CONTRIBUTIONS

All authors have contributed significantly to the manuscript. TT and NG contributed to the data acquisition. All authors contributed to the study design. CT, RJ and VG contributed to the data analysis plan, interpretation of the results and manuscript drafting. TT and NG provided expert advice and critical review of the manuscript. All authors read and approved the final version.

## Funding information

SABRE was funded at baseline by the UK Medical Research Council and Diabetes UK. Follow‐up studies have been funded by the Wellcome Trust (WT 082464), British Heart Foundation (SP/07/001/23603 and CS/13/1/30327). Metabolomic analyses were funded by Diabetes UK (13/0004774). NC received support from the National Institute for Health Research University College London Hospitals Biomedical Research Centre. Support has also been provided at follow‐up by the North and West London and Central and East London National Institute of Health Research Clinical Research Networks. VG is funded by a joint grant from Diabetes UK and the British Heart Foundation (15/0005250).

## Supporting information

Supplementary Material

## Data Availability

The data are available upon reasonable request from the SABRE Study Group. Information regarding data sharing can be found on the SABRE Study Group website (https://www.sabrestudy.org/).
