# Supplementary material for: The association between plasma metabolites and sleep quality in the Southall and Brent Revisited (SABRE) Study: A cross‐sectional analysis
Source: J Sleep Res. 2020 Dec 6;30(4):e13245. doi: 10.1111/jsr.13245 (PMC8365718; doi:10.1111/jsr.13245)
Supplement: Supplementary file 1 — Supplementary Material [file JSR-30-e13245-s001.docx]

**SUPPLEMENTARY MATERIAL FOR**:

**The association between plasma metabolites and sleep quality in the Southall and Brent Revisited Study (SABRE): A cross-sectional analysis**

**Running Head: Plasma metabolites and sleep quality in SABRE cohort**

**Authors:** Constantin-Cristian Topriceanu^1^, Therese Tillin^2,3^, Nishi Chaturvedi^2,3^, Roshni Joshi^2^, Victoria Garfield^2,3^

**Author Affiliations:**

1. University College London Medical School, 74 Huntley St, Bloomsbury, London WC1E 6DE
2. Department of Population Science and Experimental Medicine, Institute of Cardiovascular Science, University College London, Gower Street, London, WC1E 6BT, UK.
3. MRC Unit for Lifelong Health and Ageing, University College London, 1-19 Torrington Place, London, WC1E 7HB

**Corresponding author**

Constantin-Cristian Topriceanu

Medical Student

University College London

74 Huntley St, Bloomsbury,

London WC1E 6DE

Email: [zchatop@ucl.ac.uk](mailto:zchatop@ucl.ac.uk), Phone no: 020 3108 8235

**Conflict of Interest**

The views expressed in this article are those of the authors who declare that they have no conflict of interest.

**Supplementary Note 1.**

Alcohol consumption outcomes over a period of time are prone to failure when capturing differences between study participants. For example, a value of 0 could represent both structural zeros (the participant is alcohol abstinent) or random/sampling zeros (the participant is a heavy alcohol consumer but did not use any alcohol for a short period of time). Thus, the random zeros can generate an overdispersion issue which should be accounted for(He H, 2014). Of 2,718 participants, 591 (21.74%) reported having consumed zero units of alcohol justifying our use of regression models that account for zero-inflation.

**Supplementary Table S1**. Metabolic profile differences between sleep phenotype status (present/yes or absent/no) in participants from the Southall and Brent REvisited (SABRE) cohort.

|  | Yes | No |  |
| --- | --- | --- | --- |
|  | **Difficulty Falling Asleep** | |  |
| **Metabolites** |  |  | **p-value** |
| Glucose | 4.14 (3.80-4.53) | 4.15 (3.84-4.57) | 0.017 |
| Glycine | 0.29 (0.27-0.33) | 0.28 (0.26-0.32) | 0.004 |
| Glycoprotein Acetyls | 1.24 (1.13-1.39) | 1.22 (1.11-1.35) | 0.036 |
| Histidine | 0.07 (0.07-0.09) | 0.08 (0.07-0.09) | **0.0005** |
| Isoleucine | 0.06 (0.05-0.07) | 0.06 (0.05-0.07) | 0.025 |
| Leucine | 0.09 (0.09-0.10) | 0.10 (0.08-0.11) | **0.0005** |
| Sphingomyelins | 0.49 (0.46-0.52) | 0.48 (0.45-0.51) | 0.004 |
| Valine | 0.17 (0.15-0.19) | 0.18 (0.16-0.20) | **0.0004** |
| Linoleic acid | 2.79 (2.40-3.23) | 2.76 (2.36-3.14) | 0.035 |
| Free Cholesterol | 1.11 (0.95-1.28) | 1.09 (0.92-1.25) | 0.017 |
| Triglycerides in HDL | 0.11 (0.09-0.13) | 0.10 (0.09-1.25) | 0.021 |
| Triglycerides in medium HDL | 0.04 (0.03-0.05) | 0.04 (0.03-0.05) | 0.026 |
|  | **Early Morning Waking** | |  |
| Creatinine | 0.06 (0.05-0.07) | 0.06 (0.05-0.07) | 0.024 |
| Tyrosine | 0.06 (0.05-0.07) | 0.06 (0.05-0.06) | 0.046 |
| Cholesterol in medium HDL | 0.27 (0.21-0.32) | 0.27 (0.21-0.32) | 0.043 |
| Cholesterol-esters in medium HDL | 0.22 (0.18-0.27) | 0.22 (0.18-0.27) | 0.037 |
|  | **Waking Up Tired** | |  |
| Albumin | 0.09 (0.09-0.09) | 0.09 (0.09-0.09) | 0.005 |
| Lactate | 1.56 (1.39-1.81) | 1.61 (1.41-1.87) | 0.001 |
|  | **Snoring** | |  |
| Acetate | 0.07 (0.05-0.09) | 0.06 (0.05-0.08) | **0.0001** |
| Creatinine | 0.06 (0.05-0.07) | 0.06 (0.05-0.06) | **0.0001** |
| Glucose | 4.20 (3.88, 4.59) | 4.12 (3.80, 4.53) | **0.008** |
| Glutamine | 0.40 (0.28, 0.46) | 0.41 (0.31, 0.46) | **0.013** |
| Glycoprotein Acetyls | 1.23 (1.11, 1.37) | 1.22 (1.10, 1.35) | **0.002** |
| Histidine | 0.08 (0.07-0.09) | 0.08 (0.07-0.09) | 0.044 |
| Isoleucine | 0.06 (0.05-0.07) | 0.06 (0.05-0.07) | **<0.0001** |
| Lactate | 1.63 (1.43-1.87) | 1.57 (1.39-1.82) | **0.005** |
| Leucine | 0.09 (0.08-0.11) | 0.09 (0.08-0.10) | **<0.0001** |
| Sphingomyelins | 0.48 (0.45-0.51) | 0.48 (0.45-0.52) | 0.013 |
| Phosphatidylcholine | 1.57 (1.37-1.75) | 1.58 (1.40-1.78) | **0.013** |
| Phenylalanine | 0.10 (0.09, 0.11) | 0.09 (0.08, 0.10) | **<0.0001** |
| Tyrosine | 0.06 (0.05-0.07) | 0.06 (0.05-0.06) | **<0.0001** |
| Valine | 0.18 (0.16, 0.21) | 0.17 (0.15, 0.20) | **<0.0001** |
| Total cholines | 2.06 (1.84, 2.28) | 2.09 (1.87, 2.32) | **0.012** |
| Apolipoprotein A | 1.19 (1.09, 1.31) | 1.23 (1.12, 1.34) | **<0.0001** |
| Docosahexaenoic acid 22:6 | 0.14 (0.12-0.16) | 0.14 (0.12-0.17) | **0.009** |
| Linoleic acid 18:2 | 2.72 (2.33, 3.13) | 2.79 (2.40, 3.19) | **0.011** |
| Polyunsaturated fatty acids | 3.50 (2.97, 4.04) | 3.61 (3.07, 4.12) | **0.008** |
| Serum cholesterol | 3.89 (3.32, 4.50) | 3.98 (3.40, 4.58) | **0.002** |
| Serum cholesterol-esters | 2.82 (2.42, 3.25) | 2.88 (2.46, 3.32) | **0.001** |
| Free Cholesterol | 1.08 (0.91, 1.25) | 1.10 (0.94, 1.26) | **0.001** |
| Serum triglycerides | 0.92 (0.75, 1.20) | 0.92 (0.72, 1.19) | **0.002** |
| Total phosphoglycerides | 1.54 (1.35-1.73) | 1.55 (1.37-1.75) | 0.049 |
| Cholesterol in HDL | 0.99 (0.86, 1.13) | 1.02 (0.89, 1.20) | **<0.0001** |
| Cholesterol in HDL2 | 0.52 (0.41, 0.65) | 0.56 (0.44, 0.72) | **<0.0001** |
| HDL Diameter | 9.82 (9.72, 9.94) | 9.85 (9.74, 9.99) | **<0.0001** |
| Total cholesterol in small HDL | 0.34 (0.30, 0.38) | 0.35 (0.31, 0.38) | **0.004** |
| Cholesterol-esters in small HDL | 0.25 (0.21-0.29) | 0.25 (0.22-0.29) | **0.005** |
| Triglycerides in small HDL | 0.05 (0.04, 0.05) | 0.04 (0.04, 0.05) | **0.0001** |
| Total cholesterol in medium HDL | 0.26 (0.20-0.32) | 0.27 (0.21-0.33) | **0.0003** |
| Cholesterol-esters in medium HDL | 0.22 (0.17-0.26) | 0.23 (0.18-0.27) | **0.0003** |
| Free cholesterol in medium HDL | 0.04 (0.03-0.06) | 0.05 (0.03-0.06) | **0.0004** |
| Total lipids in medium HDL | 0.57 (0.47-0.68) | 0.60 (0.49-0.70) | **0.0006** |
| Concentration of Medium HDL particles | 13.8 (11.2-16.3) | 14.3 (11.7-16.7) | **0.0008** |
| Phospholipids in medium HDL | 0.28 (0.23-0.32) | 0.28 (0.24-0.33) | **0.002** |
| Total cholesterol in large HDL | 0.17 (0.21, 0.23) | 0.18 (0.13, 0.26) | **<0.0001** |
| Cholesterol-esters in large HDL | 0.14 (0.10, 0.18) | 0.15 (0.11, 0.21) | **<0.0001** |
| Free cholesterol in large HDL | 0.03 (0.02-0.05) | 0.03 (0.02-0.05) | **0.005** |
| Total lipids in large HDL | 0.35 (0.25, 0.47) | 0.38 (0.27, 0.52) | **<0.0001** |
| Concentration of large HD particles | 5.57 (4.03-7.51) | 5.99 (4.32-8.29) | **<0.0001** |
| Phospholipids in large HDL | 0.17 (0.12, 0.23) | 0.18 (0.13, 0.25) | **<0.0001** |
| Total cholesterol in very large HDL | 0.23 (0.18-0.27) | 0.23 (0.19-0.28) | **0.006** |
| Cholesterol-esters in very large HDL | 0.17 (0.13-0.20) | 0.17 (0.14-0.21) | **0.013** |
| Free cholesterol in very large HDL | 0.06 (0.04-0.07) | 0.06 (0.05-0.07) | **0.002** |
| Total lipids in very large HDL | 0.39 (0.32, 0.47) | 0.41 (0.33, 0.49) | **<0.0001** |
| Concentration of very large HDL particles | 3.75 (3.10-4.57) | 3.91 (3.17-4.74) | **0.0002** |
| Phospholipids in very large HDL | 0.16 (0.12-0.19) | 0.16 (0.13-0.21) | **<0.0001** |
| Total cholesterol in IDL | 0.70 (0.58-0.81) | 0.69 (0.57-0.81) | 0.023 |
| Cholesterol-esters in IDL | 0.50 (0.42-0.58) | 0.49 (0.41-0.58) | 0.037 |
| Free cholesterol in IDL | 0.19 (0.16, 0.22) | 0.19 (0.16, 0.23) | **0.015** |
| Total cholesterol in LDL | 1.38 (1.12, 1.67) | 1.42 (1.14, 1.73) | **0.005** |
| LDL diameter | 23.78 (23.69-23.89) | 23.79 (23.70-23.89) | **0.002** |
| Total cholesterol in small-LDL | 0.24 (0.18-0.29) | 0.24 (0.19-0.30) | **0.001** |
| Cholesterol-esters in small LDL | 0.16 (0.11-0.20) | 0.16 (0.12-0.21) | **0.003** |
| Free cholesterol in small LDL | 0.08 (0.07-0.09) | 0.08 (0.07-0.09) | **0.010** |
| Total lipids in small LDL | 0.38 (0.31-0.46) | 0.39 (0.32-0.47) | **0.006** |
| small-LDL particle concentration | 1.36 (1.09-1.63) | 1.38 (1.12-1.67) | **0.001** |
| Phospholipids in small LDL | 0.12 (0.10-0.14) | 0.12 (0.11-0.14) | **0.014** |
| Total cholesterol in medium LDL | 0.39 (0.30-0.48) | 0.40 (0.31-0.49) | **0.003** |
| Cholesterol-esters in medium LDL | 0.26 (0.19-0.34) | 0.27 (0.20-0.35) | **0.004** |
| Free cholesterol in medium LDL | 0.13 (0.11-0.14) | 0.13 (0.11-0.15) | **0.015** |
| Total lipids in medium LDL | 0.61 (0.49-0.73) | 0.62 (0.50-0.75) | 0.022 |
| Concentration of medium LDL particles | 11.9 (9.62-1.43) | 12.1 (9.69-1.46) | 0.034 |
| Triglycerides in medium LDL | 0.04 (0.03-0.05) | 0.04 (0.03-0.05) | 0.036 |
| Total cholesterol in large LDL | 0.08 (0.06-0.0.09) | 0.08 (0.06-0.09) | **0.011** |
| Cholesterol-esters in large LDL | 0.05 (0.04-0.07) | 0.06 (0.04-0.07) | 0.031 |
| Free cholesterol in large LDL | 0.02 (0.02-0.03) | 0.02 (0.02-0.0.03) | **0.020** |
| Total lipids in large LDL | 1.15 (0.96-1.35) | 1.17 (0.97-1.38) | 0.040 |
| Phospholipids in large LDL | 0.03 (0.03-0.03) | 0.03 (0.03-0.03) | 0.024 |
| Triglycerides in large LDL | 0.09 (0.07-0.12) | 0.09 (0.07-0.11) | 0.031 |
| Phospholipids in very large VLDL | 0.02 (0.01-0.04) | 0.02 (0.01-0.04) | 0.048 |
| Triglycerides in very large VLDL | 0.06 (0.04-0.10) | 0.06 (0.03-0.10) | 0.042 |

Untransformed metabolite levels values are presented as median (interquartile range). The units are mmol/l, except for the concentration of lipoprotein particles which are expressed in mol/µl, the diameter of lipoprotein particles which are expressed in nm and apolipoproteins which are expressed in g/l. Metabolite concentrations were log-transformed, mean centered and scaled to a standard deviation (SD) of 1 before further analyses. Only metabolites that were significantly different according to the t-test between sleep phenotype categories are shown.

HDL, high-density lipoprotein; IDL, intermediate-density lipoprotein; LDL, low-density lipoprotein; VLDL, very-low density lipoprotein.

P-values which remain significant at a false discovery rate of 0.05 are shown in bold.

**Supplementary Table S2**. Associations between metabolites which passed screening, and sleep phenotypes in Model 2

| **Sleep phenotype** | **Metabolite** | **Multivariable adjusted Odds Ratios** | **95% Confidence Interval** | ***p*-value** |
| --- | --- | --- | --- | --- |
| **Difficulty Falling Asleep** | Histidine | 0.89 | 0.80-0.99 | **0.033** |
|  | Leucine | 0.94 | 0.75-0.96 | **0.008** |
|  | Valine | 0.84 | 0.75-0.94 | **0.002** |
| **Snoring** | Acetate | 1.11 | 1.02-1.20 | **0.015** |
|  | Creatinine | 0.99 | 0.91-1.09 | 0.974 |
|  | Glucose | 0.98 | 0.87-1.09 | 0.705 |
|  | Glutamine | 0.96 | 0.89-1.04 | 0.314 |
|  | Glycoprotein Acetyls | 1.03 | 0.94-1.12 | 0.517 |
|  | Isoleucine | 1.15 | 1.04-1.26 | **0.004** |
|  | Lactate | 0.98 | 0.90-1.07 | 0.671 |
|  | Leucine | 1.11 | 1.01-1.21 | **0.022** |
|  | Phosphatidylcholine | 0.92 | 0.85-1.00 | 0.054 |
|  | Phenylalanine | 1.16 | 1.06-1.26 | **0.0005** |
|  | Tyrosine | 1.05 | 0.96-1.15 | 0.303 |
|  | Valine | 1.15 | 1.05-1.25 | **0.002** |
|  | Total cholines | 0.92 | 0.85-0.997 | **0.017** |
|  | Apolipoprotein A-I | 0.88 | 0.81-0.96 | **0.003** |
|  | Docosahexaenoic acid 22:6 | 0.89 | 0.82-0.96 | **0.005** |
|  | Linoleic acid 18:2 | 0.93 | 0.86-1.01 | 0.097 |
|  | Polyunsaturated fatty acids | 0.92 | 0.85-0.997 | **0.043** |
|  | Serum cholesterol | 0.90 | 0.83-0.97 | **0.006** |
|  | Serum cholesterol-esters | 0.90 | 0.83-0.96 | **0.005** |
|  | Free Cholesterol | 0.91 | 0.84-0.99 | **0.027** |
|  | Serum triglycerides | 0.95 | 0.88- 1.03 | 0.243 |
|  | Total cholesterol in HDL | 0.90 | 0.83-0.99 | **0.025** |
|  | Total cholesterol in HDL2 | 0.91 | 0.84-0.995 | **0.038** |
|  | HDL Diameter | 0.96 | 0.88-1.05 | 0.431 |
|  | Total cholesterol in small HDL | 0.91 | 0.84-0.99 | **0.022** |
|  | Cholesterol-esters in small HDL | 0.91 | 0.84-0.99 | **0.032** |
|  | Triglycerides in small HDL | 1.03 | 0.95-1.13 | 0.45 |
|  | Total cholesterol in medium HDL | 0.92 | 0.85-1.00 | 0.056 |
|  | Cholesterol-esters in medium HDL | 0.92 | 0.85-1.00 | 0.063 |
|  | Free cholesterol in medium HDL | 0.92 | 0.85-0.999 | **0.049** |
|  | Total lipids in medium HDL | 0.92 | 0.85-1.00 | 0.056 |
|  | Concentration of medium HDL particle | 0.92 | 0.85-1.00 | 0.058 |
|  | Phospholipids in medium HDL | 0.93 | 0.85-1.01 | 0.079 |
|  | Total Cholesterol in large HDL | 0.95 | 0.85-1.06 | 0.393 |
|  | Cholesterol-esters in large HDL | 0.96 | 0.89-1.05 | 0.376 |
|  | Free cholesterol in large HDL | 0.998 | 0.92-1.08 | 0.963 |
|  | Total lipids in large HDL | 0.95 | 0.88-1.04 | 0.266 |
|  | Concentration of large HDL particle | 0.95 | 0.88-1.04 | 0.27 |
|  | Phospholipids in large HDL | 0.94 | 0.87-1.03 | 0.149 |
|  | Total Cholesterol in very large HDL | 0.94 | 0.87-1.03 | 0.180 |
|  | Cholesterol-esters in very large HDL | 0.94 | 0.87-1.03 | 0.174 |
|  | Free cholesterol in very large HDL | 0.95 | 0.88-1.04 | 0.282 |
|  | Total lipids in very large HDL | 0.96 | 0.88-1.05 | 0.326 |
|  | Concentration of very large HDL particle | 0.96 | 0.88-1.05 | 0.348 |
|  | Phospholipids in very large HDL | 0.98 | 0.90-1.07 | 0.655 |
|  | Free cholesterol in IDL | 0.91 | 0.84-0.99 | **0.027** |
|  | Total cholesterol in LDL | 0.89 | 0.82-0.96 | **0.004** |
|  | LDL diameter | 1.14 | 1.05-1.23 | **0.002** |
|  | Total cholesterol in small LDL | 0.87 | 0.80-0.94 | **0.0009** |
|  | Cholesterol-esters in small LDL | 0.89 | 0.82-0.96 | **0.004** |
|  | Free cholesterol in small LDL | 0.88 | 0.81-0.96 | **0.004** |
|  | Total lipids in small LDL | 0.89 | 0.82-0.96 | **0.003** |
|  | Concentration of small LDL particle | 0.89 | 0.82-0.96 | **0.004** |
|  | Phospholipids in small LDL | 0.90 | 0.82-0.97 | **0.009** |
|  | Total Cholesterol in medium LDL | 0.89 | 0.82-0.96 | **0.002** |
|  | Cholesterol-esters in medium LDL | 0.64 | 0.45-0.92 | **0.016** |
|  | Free cholesterol in medium LDL | 0.90 | 0.82-0.97 | **0.005** |
|  | Total cholesterol in large LDL | 0.90 | 0.83-0.97 | **0.008** |
|  | Free cholesterol in large LDL | 0.90 | 0.84-0.98 | **0.02** |

All reported analyses here consisted of generalized linear mixed models with binomial distribution and logit link (i.e. logistic regression). Model 2 was adjusted for age, sex, ethnicity and years of education, waist-hip-ratio, cardiovascular disease, Type 2 Diabetes, hypertension medication, alcohol units and smoking status. Model 1 results are presented in **Table 2**. Significant p-values are highlighted in bold.

OR, odds ratio; CI, confidence interval.

Abbreviations as in **Supplementary** **Table 1.**

**Supplementary Table S3**. Associations between metabolites which passed screening and sleep phenotypes in Model 2 when using BMI instead of WHR

| **Sleep phenotype** | **Metabolite** | **Multivariable adjusted**  **Odds Ratios** | **95% Confidence Interval** | ***p*-value** |
| --- | --- | --- | --- | --- |
| **Difficulty Falling Asleep** | Histidine | 0.88 | 0.77-0.99 | **0.041** |
|  | Leucine | 0.87 | 0.77-0.99 | **0.032** |
|  | Valine | 0.87 | 0.77-0.98 | **0.018** |
| **Snoring** | Acetate | 1.11 | 1.03-1.21 | **0.009** |
|  | Creatinine | 0.97 | 0.89-1.06 | 0.505 |
|  | Glucose | 0.98 | 0.87-1.10 | 0.726 |
|  | Glutamine | 0.97 | 0.89-1.05 | 0.414 |
|  | Glycoprotein Acetyls | 1.04 | 0.96-1.13 | 0.328 |
|  | Isoleucine | 1.13 | 1.03-1.24 | **0.008** |
|  | Lactate | 0.99 | 0.91-1.08 | 0.833 |
|  | Leucine | 1.09 | 1.00-1.19 | 0.053* |
|  | Phosphatidylcholine | 0.93 | 0.86-1.01 | 0.086 |
|  | Phenylalanine | 1.16 | 1.06-1.26 | **0.001** |
|  | Tyrosine | 1.03 | 0.94-1.12 | 0.561 |
|  | Valine | 1.11 | 1.02-1.22 | **0.019** |
|  | Total cholines | 0.93 | 0.86-1.01 | 0.094* |
|  | Apolipoprotein A-I | 0.89 | 0.81-0.97 | **0.006** |
|  | Docosahexaenoic acid 22:6 | 0.86 | 0.79-0.93 | **0.0003** |
|  | Linoleic acid 18:2 | 0.94 | 0.87-1.02 | 0.144 |
|  | Polyunsaturated fatty acids | 0.92 | 0.85-1.00 | 0.050* |
|  | Serum cholesterol | 0.90 | 0.83-0.97 | **0.007** |
|  | Serum cholesterol esters | 0.89 | 0.82-0.97 | **0.006** |
|  | Free Cholesterol | 0.91 | 0.84-0.99 | **0.029** |
|  | Serum triglycerides | 0.96 | 0.88-1.04 | 0.313 |
|  | Total cholesterol in HDL | 0.91 | 0.83-0.99 | **0.034** |
|  | Total cholesterol in HDL2 | 0.92 | 0.84-0.99 | **0.047** |
|  | HDL Diameter | 0.96 | 0.88-1.05 | 0.329 |
|  | Total cholesterol in small HDL | 0.92 | 0.85-0.99 | **0.046** |
|  | Cholesterol-esters in small HDL | 0.93 | 0.86-1.01 | 0.064* |
|  | Triglycerides in small HDL | 1.04 | 0.96-1.13 | 0.350 |
|  | Total cholesterol in medium HDL | 0.93 | 0.86-1.01 | 0.102 |
|  | Cholesterol-esters in medium HDL | 0.94 | 0.86-1.02 | 0.113 |
|  | Free cholesterol in medium HDL | 0.93 | 0.86-1.01 | 0.088* |
|  | Total lipids in medium HDL | 0.94 | 0.86-1.02 | 0.113 |
|  | Concentration of medium HDL particle | 0.94 | 0.86-1.02 | 0.119 |
|  | Phospholipids in medium HDL | 0.94 | 0.87-1.02 | 0.150 |
|  | Total Cholesterol in large HDL | 0.96 | 0.88-1.04 | 0.323 |
|  | Cholesterol-esters in large HDL | 0.96 | 0.88-1.05 | 0.366 |
|  | Free cholesterol in large HDL | 1.00 | 0.92-1.08 | 0.959 |
|  | Total lipids in large HDL | 0.95 | 0.88-1.04 | 0.284 |
|  | Concentration of large HDL particle | 0.96 | 0.88-1.04 | 0.293 |
|  | Phospholipids in large HDL | 0.94 | 0.87-1.03 | 0.172 |
|  | Total Cholesterol in very large HDL | 0.93 | 0.85-1.01 | 0.088 |
|  | Cholesterol-esters in very large HDL | 0.93 | 0.85-1.01 | 0.080 |
|  | Free cholesterol in very large HDL | 0.94 | 0.87-1.01 | 0.166 |
|  | Total lipids in very large HDL | 0.94 | 0.87-1.03 | 0.177 |
|  | Concentration of very large HDL particle | 0.94 | 0.87-1.03 | 0.192 |
|  | Phospholipids in very large HDL | 0.97 | 0.89-1.05 | 0.456 |
|  | Free cholesterol in IDL | 0.91 | 0.84-0.99 | **0.028** |
|  | Total cholesterol in LDL | 0.89 | 0.82-0.97 | **0.005** |
|  | LDL diameter | 1.13 | 1.04-1.23 | **0.003** |
|  | Total cholesterol in small LDL | 0.87 | 0.80-0.95 | **0.001** |
|  | Cholesterol-esters in small LDL | 0.89 | 0.82-0.97 | **0.005** |
|  | Free cholesterol in small LDL | 0.88 | 0.81-0.96 | **0.005** |
|  | Total lipids in small LDL | 0.89 | 0.82-0.96 | **0.004** |
|  | Concentration of small LDL particle | 0.89 | 0.82-0.97 | **0.005** |
|  | Phospholipids in small LDL | 0.90 | 0.83-0.98 | **0.014** |
|  | Total Cholesterol in medium LDL | 0.89 | 0.82-0.96 | **0.003** |
|  | Cholesterol-esters in medium LDL | 0.65 | 0.45-0.93 | **0.017** |
|  | Free cholesterol in medium LDL | 0.90 | 0.83-0.97 | **0.008** |
|  | Total cholesterol in large LDL | 0.90 | 0.83-0.97 | **0.010** |
|  | Free cholesterol in large LDL | 0.91 | 0.84-0.99 | **0.024** |

* Difference in significance Model 2 with body mass index and Model 2 with waist-to-hip-ratio.

All reported analyses here consisted of generalized linear mixed models with binomial distribution and logit link (i.e. logistic regression). Model 2 was adjusted for age, sex, ethnicity and years of education, body mass index, cardiovascular disease, Type 2 Diabetes, hypertension medication, alcohol units and smoking status.

OR, odds ratio; CI, confidence interval.

Abbreviations as in **Supplementary** **Table 1.**

**Supplementary Table S4**. Plasma levels of metabolites which passed screening, by sleep quality phenotype

| **Sleep phenotype** | **Metabolite** | **Sleep Phenotype Present** | **Sleep Phenotype Absent** | ***p*-value** |
| --- | --- | --- | --- | --- |
| **Difficulty Falling Asleep** | Histidine | 0.07 (0.07, 0.09) | 0.08 (0.07, 0.09) | **0.013** |
|  | Leucine | 0.09 (0.09, 0.10) | 0.10 (0.8, 0.11) | **0.004*** |
|  | Valine | 0.17 (0.15, 0.19) | 0.18 (0.16, 0.20) | **0.001** |
| **Snoring** | Acetate | 0.07 (0.05, 0.09) | 0.06 (0.05, 0.08) | **0.0001** |
|  | Creatinine | 5.89 *10^-2^  (5.22*10^-2^, 6.62*10^-2^) | 5.76 *10^-2^  (5.08*10^-2^, 6.48*10^-2^) | **0.0002*** |
|  | Glucose | 4.20 (3.88, 4.59) | 4.12 (3.80, 4.53) | **0.001*** |
|  | Glutamine | 0.40 (0.28, 0.46) | 0.41 (0.31, 0.46) | **0.022*** |
|  | Glycoprotein Acetyls | 1.23 (1.11, 1.37) | 1.22 (1.10, 1.35) | **0.005** |
|  | Isoleucine | 5.93*10^-2^  (5.19*10^-2^, 6.80*10^-2^) | 5.67*10^-2^  (4.82*10^-2^, 6.50*10^-2^) | **<0.0001** |
|  | Lactate | 1.63 (1.43, 1.87) | 1.57 (1.39, 1.82) | **0.001*** |
|  | Leucine | 9.28*10^-2^  (8.21*10^-2^, 10.73*10^-2^) | 8.91*10^-2^  (7.89*10^-2^, 10.27*10^-2^) | **<0.0001** |
|  | Phosphatidylcholine | 1.57 (1.37, 1.75) | 1.58 (1.40, 1.78) | **0.028*** |
|  | Phenylalanine | 0.10 (0.09, 0.11) | 0.09 (0.08, 0.10) | **<0.0001** |
|  | Tyrosine | 5.80 *10^-2^  (5.03*10^-2^, 6.55*10^-2^) | 5.57 *10^-2^  (4.93*10^-2^, 6.35*10^-2^) | **<0.0001** |
|  | Valine | 0.18 (0.16, 0.21) | 0.17 (0.15, 0.20) | **<0.0001** |
|  | Total cholines | 2.06 (1.84, 2.28) | 2.09 (1.87, 2.32) | **0.028*** |
|  | Apolipoprotein A-I | 1.19 (1.09, 1.31) | 1.23 (1.12, 1.34) | **<0.0001** |
|  | Docosahexaenoic acid 22:6 | 13.67 *10^-2^  (11.76*10^-2^, 16.14*10^-2^) | 14.00 *10^-2^  (11.86*10^-2^, 16.67*10^-2^) | **0.049** |
|  | Linoleic acid 18:2 | 2.72 (2.33, 3.13) | 2.79 (2.40, 3.19) | **0.002*** |
|  | Polyunsaturated fatty acids | 3.50 (2.97, 4.04) | 3.61 (3.07, 4.12) | **0.001*** |
|  | Serum cholesterol | 3.89 (3.32, 4.50) | 3.98 (3.40, 4.58) | **0.0006** |
|  | Serum Cholesterol-esters | 2.82 (2.42, 3.25) | 2.88 (2.46, 3.32) | **0.005** |
|  | Free Cholesterol | 1.08 (0.91, 1.25) | 1.10 (0.94, 1.26) | **0.026** |
|  | Serum triglycerides | 0.92 (0.75, 1.20) | 0.92 (0.72, 1.19) | 0.179 |
|  | Total cholesterol in HDL | 0.99 (0.86, 1.13) | 1.02 (0.89, 1.20) | **<0.0001** |
|  | Total cholesterol in HDL2 | 0.52 (0.41, 0.65) | 0.56 (0.44, 0.72) | **<0.0001** |
|  | HDL Diameter | 9.82 (9.72, 9.94) | 9.85 (9.74, 9.99) | **<0.0001** |
|  | Total cholesterol in small HDL | 0.34 (0.30, 0.38) | 0.35 (0.31, 0.38) | **0.016** |
|  | Cholesterol-esters in small HDL | 24.78 *10^-2^  (20.80*10^-2^, 28.79*10^-2^) | 25.42 *10^-2^  (21.87*10^-2^, 28.81*10^-2^) | **0.0009** |
|  | Triglycerides in small HDL | 0.05 (0.04, 0.05) | 0.04 (0.04, 0.05) | **0.0001** |
|  | Total cholesterol in medium HDL | 0.26 (0.21, 0.32) | 0.27 (0.21, 0.33) | **0.0001** |
|  | Cholesterol-esters in medium HDL | 0.22 (0.17, 0.26) | 0.23 (0.18, 0.27) | **<0.0001** |
|  | Free cholesterol in medium HDL | 0.04 (0.03, 0.06) | 0.05 (0.03, 0.06) | **0.0002** |
|  | Total lipids in medium HDL | 0.57 (0.47, 0.68) | 0.60 (0.49, 0.70) | **0.0006*** |
|  | Concentration of medium HDL particle | 1.38 *10^-6^  (1.12*10^-6^, 1.63*10^-6^) | 1.43 *10^-6^  (1.17*10^-6^, 1.67*10^-6^) | **0.0009*** |
|  | Phospholipids in medium HDL | 27.51 *10^-2^  (22.65*10^-2^, 32.19*10^-2^) | 28.33 *10^-2^  (23.55*10^-2^, 32.97*10^-2^) | **0.002*** |
|  | Total Cholesterol in large HDL | 0.17 (0.21, 0.23) | 0.18 (0.13, 0.26) | **<0.0001*** |
|  | Cholesterol-esters in large HDL | 0.14 (0.10, 0.18) | 0.15 (0.11, 0.21) | **<0.0001** |
|  | Free cholesterol in large HDL | 2.97 *10^-2^  (1.78*10^-2^, 4.58*10^-2^) | 3.28 *10^-2^  (1.98*10^-2^, 5.20*10^-2^) | **<0.0001*** |
|  | Total lipids in large HDL | 0.35 (0.25, 0.47) | 0.38 (0.27, 0.52) | **<0.0001** |
|  | Concentration of large HDL particle | 5.57 *10^-7^  (4.03*10^-7^, 7.51*10^-7^) | 5.99 *10^-7^  (4.32*10^-7^, 8.29*10^-7^) | **<0.0001** |
|  | Phospholipids in large HDL | 0.17 (0.12, 0.23) | 0.18 (0.13, 0.25) | **<0.0001** |
|  | Total Cholesterol in very large HDL | 22.51*10^-2^  (17.49*10^-2^, 27.22*10^-2^) | 23.16*10^-2^  (18.53*10^-2^, 27.67*10^-2^) | **0.010** |
|  | Cholesterol-esters in very large HDL | 16.85*10^-2^  (13.20*10^-2^, 20.37*10^-2^) | 17.27*10^-2^  (13.87*10^-2^, 20.64*10^-2^) | **0.027** |
|  | Free cholesterol in very large HDL | 5.53*10^-2^  (4.16*10^-2^, 6.95*10^-2^) | 5.82*10^-2^  (4.48*10^-2^, 7.23*10^-2^) | **0.001** |
|  | Total lipids in very large HDL | 0.39 (0.32, 0.47) | 0.41 (0.33, 0.49) | **0.0007** |
|  | Concentration of very large HDL particle | 3.75*10^-7^  (3.10*10^-7^, 4.57*10^-7^) | 3.91 *10^-7^  (3.17*10^-7^, 4.74*10^-7^) | **0.0007** |
|  | Phospholipids in very large HDL | 15.62*10^-2^  (12.40*10^-2^, 19.39*10^-2^) | 16.22*10^-2^  (12.59*10^-2^, 20.91*10^-2^) | **0.0004** |
|  | Free cholesterol in IDL | 0.19 (0.16, 0.22) | 0.19 (0.16, 0.23) | 0.059* |
|  | Total cholesterol in LDL | 1.38 (1.12, 1.67) | 1.42 (1.14, 1.73) | **0.038** |
|  | LDL diameter | 23.80 (23.71, 23.91) | 23.78 (23.69, 23.89) | **0.009** |
|  | Total cholesterol in small LDL | 23.54 *10^-2^  (18.07*10^-2^, 28.93*10^-2^) | 24.19 *10^-2^  (18.92*10^-2^, 30.12*10^-2^) | **0.010** |
|  | Cholesterol-esters in small LDL | 15.59 *10^-2^  (11.38*10^-2^, 20.04*10^-2^) | 16.23 *10^-2^  (12.12*10^-2^, 21.15*10^-2^) | **0.007** |
|  | Free cholesterol in small LDL | 7.79 *10^-2^  (6.57*10^-2^, 8.89*10^-2^) | 7.89 *10^-2^  (6.74*10^-2^, 9.06*10^-2^) | 0.053* |
|  | Total lipids in small LDL | 0.38 (0.31, 0.46) | 0.39 (0.32, 0.47) | **0.035** |
|  | Concentration of small LDL particle | 1.36 *10^-7^  (1.09*10^-7^, 1.63*10^-7^) | 1.38 *10^-7^  (1.12*10^-7^, 1.67*10^-7^) | **0.048** |
|  | Phospholipids in small LDL | 12.11 *10^-2^  (10.35*10^-2^, 13.84*10^-2^) | 12.24 *10^-2^  (10.63*10^-2^, 14.02*10^-2^) | 0.052* |
|  | Total Cholesterol in medium LDL | 0.39 (0.30, 0.48) | 0.40 (0.31, 0.49) | **0.025** |
|  | Cholesterol-esters in medium LDL | 0.26 (0.19, 0.34) | 0.27 (0.20, 0.35) | **0.020** |
|  | Free cholesterol in medium LDL | 12.63 *10^-2^  (10.83*10^-2^, 14.36*10^-2^) | 12.72*10^-2^  (10.98*10^-2^, 14.57*10^-2^) | 0.081* |
|  | Total cholesterol in large LDL | 7.64*10^-2^  (6.30*10^-2^, 9.09*10^-2^) | 7.79*10^-2^  (6.33*10^-2^, 9.31*10^-2^) | 0.079* |
|  | Free cholesterol in large LDL | 22.37 *10^-2^  (19.16*10^-2^, 25.83*10^-2^) | 22.70*10^-2^  (19.21*10^-2^, 26.46*10^-2^) | 0.079* |

* Difference in significance between Wilcoxon ranked sum test and generalized linear mixed models with binomial distribution and logit link in Model 1.

Values are presented as median (interquartile range). P-values were calculated using Wilcoxon ranked sum test with continuity correction. Significant p-values are highlighted in bold.

The units are mmol/l, except for concentration of lipoprotein particles which are expressed in mol/l, the diameter of lipoprotein particles which are expressed in nm and apolipoproteins which are expressed in g/l.

Abbreviations as in **Supplementary** **Table 1.**

**Supplementary Table S5**. Associations between metabolites and sleep quality phenotypes in the sensitivity analysis

|  |  | **Model 2** | |
| --- | --- | --- | --- |
| **Sleep phenotype** | **Metabolite** | **OR (95% CI)** | ***p*-value** |
| **Difficulty Falling Asleep** | Glycine | 1.15 (1.04-1.27) | **0.008** |
|  | Free cholesterol | 1.13 (1.02-1.26 | **0.025** |
|  | Sphingomyelins | 1.15 (1.03-1.15) | **0.010** |
| **Early Morning Waking** | Creatinine | 0.91 (0.83-0.99) | **0.029** |
|  | Valine | 0.90 (0.82-0.98 | **0.013** |
| **Waking Up Tired** | Albumin | 0.88 (0.81-0.96) | **0.002** |
|  | Lactic acid | 0.86 (0.78-0.94) | **0.0006** |
| **Snoring** | Apolipoprotein B | 0.91 (0.84-0.99) | **0.022** |
|  | Serum cholesterol-esters | 0.90 (0.83-0.98) | **0.014** |
|  | Total cholesterol in IDL | 0.92 (0.85-0.99) | **0.037** |
|  | Free cholesterol in large LDL | 0.91 (0.84-0.99) | **0.020** |
|  | Total lipids in large LDL | 0.91 (0.84-0.98) | **0.016** |
|  | Concentration of large LDL particles | 0.91 (0.84-0.99) | **0.019** |
|  | Phospholipids in large LDL | 0.91 (0.84-0.98) | **0.016** |
|  | Total lipids in medium LDL | 0.90 (0.83-0.97) | **0.008** |
|  | Concentration of medium LDL particles | 0.90 (0.83-0.98) | **0.010** |
|  | Phospholipids in medium LDL | 0.91 (0.84-0.98) | **0.019** |
|  | Free cholesterol in small VLDL | 0.93 (0.85-0.999) | **0.048** |
|  | Phospholipids in small VLDL | 0.91 (0.84-0.99) | **0.030** |
|  | Free cholesterol in very small VLDL | 0.91 (0.84-0.98) | **0.019** |
|  | Phospholipids in very small VLDL | 0.92 (0.85-0.99) | **0.035** |

Only metabolites that were significant in the fully adjusted Model 2, but did not pass the screening stage are shown here.

All reported analyses here consisted of generalized linear mixed models with binomial distribution and logit link (i.e. logistic regression). Model 2 was adjusted for age, sex, ethnicity and years of education, waist-hip-ratio, cardiovascular disease, Type 2 Diabetes, hypertension medication, alcohol units and smoking status.

Abbreviations as in **Supplementary Table S2.**

**Supplementary Table S6**. Associations between metabolites and the composite sleep score (wSleep)

| **Metabolite** | **Exponentiated Estimate (95% CI)** | ***p*-value** |
| --- | --- | --- |
| Glycoprotein acetyls | 1.04 (1.00-1.08) | **0.029** |
| Sphingomyelins | 1.05 (1.01-1.09) | **0.008** |
| Serum triglycerides | 1.04 (1.01-1.08) | **0.025** |
| Triglycerides in s-LDL | 1.04 (1.00-1.08) | **0.049** |
| Triglycerides in m-LDL | 1.04 (1.00-1.08) | **0.043** |
| Triglycerides in s-VLDL | 1.04 (1.01-1.08) | **0.024** |
| Free Cholesterol in m-VLDL | 1.04 (1.00-1.08) | **0.036** |
| Total lipids in m-VLDL | 1.04 (1.00-1.08) | **0.041** |
| Phospholipids in m-VLDL | 1.04 (1.00-1.08) | **0.044** |
| Triglycerides in m-VLDL | 1.04 (1.01-1.08) | **0.026** |
| Diameter of VLDL | 1.04 (1.00-1.07) | **0.033** |
| Triglycerides in VLDL | 1.04 (1.01-1.08) | **0.023** |
| Total lipids in l-VLDL | 1.04 (1.00-1.08) | **0.047** |
| Triglycerides in l-VLDL | 1.04 (1.00-1.08) | **0.032** |
| Triglycerides in xl-VLDL | 1.03 (1.01-1.07) | **0.016** |

All reported analyses here consisted of generalized linear mixed models with poison distribution and log link. Model 1 was adjusted for age, sex, ethnicity and years of education. Only metabolites that were significant in Model 1 are shown.

**Supplementary Figure S1.** Correlation matrix of HDL and LDL sub-fractions

Positive correlations are displayed in blue and negative correlations are displayed in red. Color intensity and the size of the circle is proportional to correlation coefficients. The legend color displays the correlation coefficients range and the corresponding colors.

HDL, high-density lipoprotein; s-HDL, small-HDL; m-HDL, medium-HDL; l-HDL, large-HDL, xl-HDL, extra-large HDL; IDL, intermediate-density lipoprotein; LDL, low-density lipoprotein; s-LDL, small-LDL; m-LDL, medium-LDL; l-LDL, large-LDL; CE, cholesterol-esters; FC, free cholesterol;

**REFERENCES:**

He H, W. W., Crits-Christoph P, Gallop R, Tang W, Chen DD, Tu XM. (2014). On the  implication  of  structural   zeros  as  independent   variables  in  regression   analysis :  applications  to  alcohol research. In (Vol. 12(3), pp. 439-460). J Data Sci.
